# Objectively Measured Intensity-specific Physical Activity and Hippocampal Volume Among Community-dwelling Older Adults

**DOI:** 10.2188/jea.JE20200534

**Published:** 2022-11-05

**Authors:** Masaki Machida, Tomoko Takamiya, Shiho Amagasa, Hiroshi Murayama, Takeo Fujiwara, Yuko Odagiri, Hiroyuki Kikuchi, Noritoshi Fukushima, Mitsuo Kouno, Yu Saito, Fumitoshi Yoshimine, Shigeru Inoue, Yugo Shobugawa

**Affiliations:** 1 Department of Preventive Medicine and Public Health, Tokyo Medical University, Tokyo, Japan; 2 Research Team for Social Participation and Community Health, Tokyo Metropolitan Institute of Gerontology, Tokyo, Japan; 3 Department of Global Health Promotion, Tokyo Medical and Dental University, Tokyo, Japan; 4 Niigata Prefectural Tokamachi Hospital, Niigata, Japan; 5 Department of Active Ageing (donated by Tokamachi city, Niigata Japan), Niigata University Graduate School of Medical and Dental Sciences, Niigata, Japan

**Keywords:** epidemiology, accelerometry, aging, hippocampus, compositional data analysis

## Abstract

**Background:**

The hippocampus is a brain structure important for memory and cognitive function. Physical activity may help prevent hippocampal atrophy. However, few studies have measured sedentary behavior (SB) and intensity-specific physical activity using an accelerometer. This study aimed to examine the cross-sectional associations of objectively-determined SB, light-intensity physical activity (LPA), and moderate-to-vigorous physical activity (MVPA) measured by an accelerometer with hippocampal volume among community-dwelling older adults using compositional data analysis (CoDa) approach.

**Methods:**

This cross-sectional study was part of the Neuron to Environmental Impact across Generations (NEIGE) study. A randomly recruited sample of 485 Japanese older adults (47% male; aged 65–84 years) wore tri-axial accelerometers (Omron Healthcare) for 7 consecutive days in 2017. Hippocampal volume was measured with magnetic resonance imaging and the left and right hippocampal volumes were automatically segmented using FreeSurfer software. Associations of sedentary and physically active behaviors with hippocampal volume were examined with compositional linear regression analysis based on isometric log-ratio transformations of time use adjusted for potential confounding factors.

**Results:**

The relative proportion of time spent in MVPA, compared to the other two activities, was significantly positively associated with right hippocampal volume (β: 57.1, *P*-value = 0.027). However, no association existed between higher proportions of MVPA and left hippocampal volume, or between proportions of SB or LPA with either left or right hippocampal volumes.

**Conclusion:**

The proportion of time spent in MVPA, relative to the other two activities, was significantly positively associated with right hippocampal volume. MVPA may be beneficial for maintaining hippocampal volume.

## INTRODUCTION

The incidence of dementia is increasing globally and has become a major public health concern.^1^ A meta-analysis of the global literature on the prevalence of dementia revealed that there were 35.6 million people with dementia across the world in 2010. This number nearly doubles every 20 years and is estimated to reach 65.7 million in 2030 and 115.4 million in 2050.^2^ Effective preventive strategies are, therefore, urgently required.

The hippocampus is a structure within the brain that has major roles in memory and cognitive function.^3^ Individuals with greater hippocampal volumes have a lower risk of cognitive impairment,^4^ and among patients with mild cognitive impairment (MCI), a smaller hippocampal volume increases the risk of progression to Alzheimer’s-type dementia.^5^ It is expected that strategies which prevent hippocampal atrophy would be effective for the prevention of dementia.

Some studies have focused on the relationship between physical activity and hippocampal volume.^4^^,^^6^^–^^10^ However, these previous studies have several shortcomings. First, in several previous studies, the participants were volunteers, who consisted of only cognitively normal older adults.^4^^,^^6^^,^^7^^,^^9^ People who volunteer as study participants tend to be healthier than those who do not, so they may not be representative of the intended target population.^11^

Second, several previous studies measured physical activity using questionnaires,^4^^,^^6^^,^^7^ which have a lower validity than other objective measures of physical activity.^12^ Although an accelerometer can determine activity behaviors more accurately than questionnaires and can also measure the intensity level of physical activity,^12^ only a few studies have applied this method to investigate the relationship between physical activity and hippocampal volume.^8^^–^^10^ Furthermore, few have clarified this relationship according to the intensity level of physical activity, such as sedentary behavior (SB), light-intensity physical activity (LPA), and moderate-to-vigorous physical activity (MVPA).^8^^,^^10^

Third, the statistical methods applied in previous studies may be misleading. Time is finite during the day, and SB, LPA, and MVPA are not mutually independent. For example, if the time spent in MVPA increases or decreases, this can influence the time spent in SB and LPA. Conventional statistical methods can be misleading, overestimating, or underestimating some effects. Compositional data analysis (CoDa) allows for the consideration of the co-dependence of the time spent in all behaviors throughout the day or during another fixed period.^13^^,^^14^ However, to the best of our knowledge, the only study that examined the association of physical activity and hippocampal volume using the CoDa approach is a recent study conducted among children.^10^

Therefore, given the above points, we examined the associations of objectively determined SB, LPA, and MVPA with hippocampal volume in randomly recruited community-dwelling older adults using a CoDa approach.

## METHODS

### Study sample and data collection

This cross-sectional study was a part of the Neuron to Environmental Impact across Generations (NEIGE) study.^15^ Participants were community-dwelling older adults who did not require long-term care and resided in the city of Tokamachi, Niigata Prefecture, Japan. Tokamachi is a rural city located in the southernmost region of the Niigata Prefecture (area: 590.4 km^2^, population: 51,964, as of January 31, 2020). Altogether, 1,346 residents (aged 65–84 years) were selected from a resident registry, using stratified random sampling (stratification factors: age and residential area).^15^ In the fall of 2017, 527 participants who agreed to enroll in the NEIGE study completed a questionnaire and a health examination, and at the same time consented to wear an accelerometer and undergo magnetic resonance imaging (MRI) (response rate: 39.2%). A detailed profile of this study has been reported elsewhere.^15^

Ethical approval was granted by the University Ethics Committees of Niigata University and Tokyo Medical University (approval numbers 2666 and 3921). Written informed consent was obtained from all participants.

### Assessment of activity behaviors

Participants were instructed to wear an accelerometer, the Active style Pro HJA-750C (Omron Healthcare, Kyoto, Japan), over the waist on an elasticated belt for 7 consecutive days while awake except during water-based activities (eg, swimming and hot springs) between September 2017 and October 2017. Active style Pro is a validated accelerometer^16^^–^^18^ that provides data comparable to devices most commonly used in studies conducted in Western countries.^19^^,^^20^ Its algorithm has been explained in detail elsewhere.^16^^,^^17^ The absence of an acceleration signal for longer than 60 consecutive minutes was defined as “non-wear,” and records from participants wearing the accelerometer for at least 10 hours per day were considered valid.^21^ Participants with 4 or more valid days of valid data were included in the analyses.^22^ We used 60-second epochs of data to obtain estimated metabolic equivalents (METs) values using analysis software. METs-based criteria were used to categorize behavior into activities of various intensities: ≤1.5 METs: SB, 1.6–2.9 METs: LPA, and ≥3.0 METs: MVPA.^23^^,^^24^ The analyses included the sub-compositions of activity behaviors that constitute accelerometer-wearing time (SB, LPA, and MVPA).

### Assessment of hippocampal volume

Brain images were acquired using a 1.5 Tesla MRI scanner (MAGNETOM Avanto fit, Siemens, Germany) using the three-dimensional magnetization-prepared rapid gradient-echo sequence with the following parameters: repetition time = 1,700 ms; echo time = 4.31 ms; inversion time = 800 ms; flip angle = 15°; 144 contiguous 1.25 mm slices; field of view = 230 × 230; matrix size = 256 × 256. Segmentation of the hippocampus and volume calculations were performed using FreeSurfer Version 6.0 (http://surfer.nmr.mgh.harvard.edu). Briefly, the automated FreeSurfer protocol first included removal of non-brain tissue, labeling volumes of each segmentation, and normalizing the voxel intensities. Next, cortical and subcortical volume measures were derived using the surface stream and the subcortical segmentation stream, respectively. Detailed methods for volume derivation have been explained in detail elsewhere.^25^^–^^27^ Right and left hippocampal volume and intracranial volume (ICV) were automatically derived from the subcortical processing stream (ie, “aseg.stats” in FreeSurfer). Quality checks of acquired data were conducted manually.

### Covariates and other variables

Participants reported their sex, age, living arrangement (with others or alone), working status (worker or non-worker), educational attainment (<13 years or ≥13 years), smoking status (smoker or non-smoker), and alcohol use (<60 g or ≥60 g of ethanol/day). Medical professionals collected information regarding the use of medication for hypertension, dyslipidemia, and diabetes through an interview. Body mass index (BMI) was calculated using height and weight (kg/m^2^) measurements taken with a body composition analyzer MC-780A (TANITA Corporation, Tokyo, Japan).

### Statistical analyses

R version 3.6.2 (R Foundation for Statistical Computing, Vienna, Austria) was used to perform all statistical analyses. We employed the CoDa approach detailed in a previous study^13^ using “compositions” from the R package. Statistical significance was set at *P* < 0.05. We generated compositional data by dividing the time spent performing each behavior (SB, LPA, and MVPA) by the total accelerometer wear time. The compositional data of SB, LPA, and MVPA were defined as T_SB_, T_LPA_, and T_MVPA_, respectively. The sum of T_SB_, T_LPA_, and T_MVPA_ for each individual is 1. Then, to clarify the codependence between components (T_SB_, T_LPA_, and T_MVPA_), we created a variation matrix, which is a symmetric matrix that contains all the possible log-ratios.^13^^,^^28^ A value close to 0 implies that the time spent in the corresponding behaviors was nearly proportional, hence, there is a high co-dependence between them. To investigate associations between activity behaviors and left and right hippocampal volume, we applied a compositional multiple linear regression analysis using log-ratio transformations of the time-use composition.^13^ The isometric log-ratio (ilr) transform maps compositional data from simple space to real space, and preserves all metric data properties.^29^ For example, when we analyzed the association of MVPA with the right hippocampal volume, the ilr transformations of compositional data from the three activities (T_SB_, T_LPA_, and T_MVPA_) were entered into the regression model and adjusted for potential confounding factors. We obtained the results for the first ilr transformations for T_SB_, T_LPA_, and T_MVPA_ using the following formula:

$$z_{1}=\sqrt{\frac{2}{3}}\ln\frac{T_{\text{MVPA}}}{\sqrt{T_{\text{SB}}\cdot T_{\text{LPA}}}}$$

$$z_{2}=\sqrt{\frac{1}{2}}\ln\frac{T_{\text{LPA}}}{T_{\text{SB}}}$$

Subsequently, we performed a compositional multiple linear regression analysis with Z1 and Z2 as independent variables. The beta of Z1, calculated in this analysis, shows the association between the relative proportion of MVPA and right hippocampal volume, compared to the other two activities. In this study, model 1 was unadjusted. Model 2 was adjusted for sex, age, and ICV. Model 3 was adjusted for model 2 plus educational attainment. Model 4 was adjusted for model 3 plus BMI (<18.5, 18.5–24.9, or ≥25.0 kg/m^2^), smoking status, and alcohol use. Model 5 was adjusted for model 4 plus the use of medication for hypertension, dyslipidemia, and diabetes. These covariates were determined according to previous research methodology for investigations of physical activity and hippocampal volume.^4^^,^^8^^–^^10^ If activity behaviors were observed to be significantly associated with hippocampal volume, we estimated the change in hippocampal volume when fixed-length sections of time were reallocated from one part of a particular composition to another, while remaining parts were kept constant.^30^^,^^31^

In volumetric neuroimaging studies, another method of statistically adjusting for ICV (which is a covariate of hippocampal volume) is to use the value of the hippocampal volume divided by the ICV (we refer to this as “the proportion of hippocampal volume”) as a dependent variable.^32^ Because variations in statistical adjustment methods may lead to different results,^32^ we also applied compositional multiple linear regression analysis using ilr transformations of time-use composition with the proportion of hippocampal volume as the dependent value, adjusting for other potential confounding factors.

## RESULTS

### Participant enrollment and characteristics

Of the 527 older adults who consented to wear an accelerometer and undergo MRI scans (response rate: 39.2%), 42 were excluded. Exclusion criteria included: failure to wear the accelerometer for the required time to meet the criteria (*n* = 14), accelerometer system error (*n* = 1), and failure to undergo MRI scan (*n* = 27). The final sample used for analyses in this study included 485 participants. The participant characteristics are presented in Table 1.

**Table 1.** Participants’ characteristics

	Participants: *n* = 485			

	*n*	(%)	mean	(SD)
Sex, male	228	(47.0%)		
Age, years			73.3	(5.5)
Body mass index, kg/m^2^				
Mean			22.5	(3.0)
<18.5, kg/m^2^	40	(8.2%)		
≥25.0, kg/m^2^	86	(17.7%)		
Education, ≥13 years	95	(19.6%)		
Living arrangement, with others	442	(91.1%)		
Working status, worker	235	(48.5%)		
Smoking status, smoker	41	(8.5%)		
Alcohol use, ≥60 g of ethanol/day	22	(4.5%)		
Use of medication, yes				
Hypertension	221	(45.6%)		
Dyslipidemia	167	(34.4%)		
Diabetes	46	(9.5%)		
Accelerometer wear time, min/day			887.5	(109.4)
Activity time, arithmetic mean				
Sedentary behavior (SB), min/day			442.7	(129.6)
Light-intensity physical activity (LPA), min/day			391.2	(100.9)
Moderate-to-vigorous physical activity (MVPA), min/day			53.6	(40.2)
Brain volume, arithmetic mean				
Intracranial volume, mm^3^			1,432,369.0	(152,092.0)
Right hippocampal volume, mm^3^			3,687.7	(420.3)
Left hippocampal volume, mm^3^			3,522.1	(391.1)

SD, standard deviation.

Table 2 presents the variation matrix indicating the dispersion of each behavior. The highest log-ratio variances all involved MVPA, which indicated that the time spent in MVPA is the least co-dependent on the other behaviors. The largest variability was in the ratio of MVPA to SB.

**Table 2.** Variation matrix of time spent in activities

	SB	LPA	MVPA
SB	0		
LPA	0.26	0	
MVPA	1.19	0.76	0

LPA, light-intensity physical activity; MVPA, moderate-to-vigorous physical activity; SB, sedentary behavior.

A value close to zero implies that the times spent in the behaviors involved in the ratio are highly proportional.

### Associations between sedentary and physically active behaviors and hippocampal volume

The results of the multiple linear regression models of left and right hippocampal volumes are presented in Table 3. In all models, the relative proportion of time spent in MVPA, compared to the other two activities, was significantly positively associated with the right hippocampal volume [model 5; β: 57.1, 95% confidence interval (CI), 6.5–107.6, *P*-value = 0.027]. The proportions of time spent engaging in LPA and SB relative to the other behaviors were not associated with right hippocampal volume in any adjusted model. No associations were observed between the left hippocampal volume and the relative proportion of time spent engaging in any behavior in any adjusted model. When the dependent variable was the proportion of hippocampal volume, only the relative proportion of time spent in MVPA, compared to the other two activities, displayed a statistically significant association with the proportion of the right hippocampal volume (model 5; β: 48.6 × 10^−6^, *P*-value = 0.014). No association was observed between the relative proportion of time spent in MVPA and the proportion of left hippocampal volume, or between SB/LPA and left/right hippocampal volume.

**Table 3.** Associations between sedentary and physically active behaviors and hippocampal volume in older adults

Sedentary and physically active behaviors	SB		LPA		MVPA	

	β (95% CI)	*P*	β (95% CI)	*P*	β (95% CI)	*P*
Right hippocampal volume						
Model 1	59.9 (−29.8–149.6)	0.190	−220.5 (−333.1–−107.9)	<0.001	160.6 (108.4–212.8)	<0.001
Model 2	−6.4 (−88.7–75.9)	0.878	−44.3 (−148.4–59.9)	0.404	50.7 (1.4–100.0)	0.044
Model 3	−11.5 (−94.0–71.1)	0.785	−40.3 (−144.5–63.9)	0.447	51.8 (2.5–101.1)	0.039
Model 4	−12.3 (−95.1–70.5)	0.771	−46.7 (−151.4–58.1)	0.382	58.9 (8.5–109.4)	0.022
Model 5	−22.7 (−106.2–60.8)	0.593	−34.3 (−139.5–70.8)	0.522	57.1 (6.5–107.6)	0.027

Left hippocampal volume						
Model 1	113.7 (29.4–198.0)	0.008	−232.8 (−338.7–−126.9)	<0.001	119.1 (70.0–168.2)	<0.001
Model 2	43.0 (−33.6–119.5)	0.271	−51.4 (−148.2–45.5)	0.298	8.4 (−37.5–54.2)	0.720
Model 3	39.6 (−37.3–116.4)	0.312	−48.7 (−145.8–48.3)	0.324	9.2 (−36.7–55.0)	0.695
Model 4	38.6 (−38.3–115.4)	0.324	−58.6 (−155.9–38.7)	0.237	20.0 (−26.8–66.8)	0.402
Model 5	34.9 (−42.8–112.6)	0.378	−52.4 (−150.2–45.5)	0.294	17.5 (−29.6–64.5)	0.466

CI, confidence interval; LPA, light-intensity physical activity; MVPA, moderate-to-vigorous physical activity; SB, sedentary behavior.

Model 1: unadjusted model, Model 2: adjusted for sex, age, and intracranial volume, Model 3: adjusted for model 2 plus education, Model 4: adjusted for model 3 plus body mass index, smoking status, and alcohol use, Model 5: adjusted for model 4 plus medication for hypertension, dyslipidemia, and diabetes.

Isometric log-ratio (ilr) transformation was used for compositional regression analysis.

**Figure 1.**
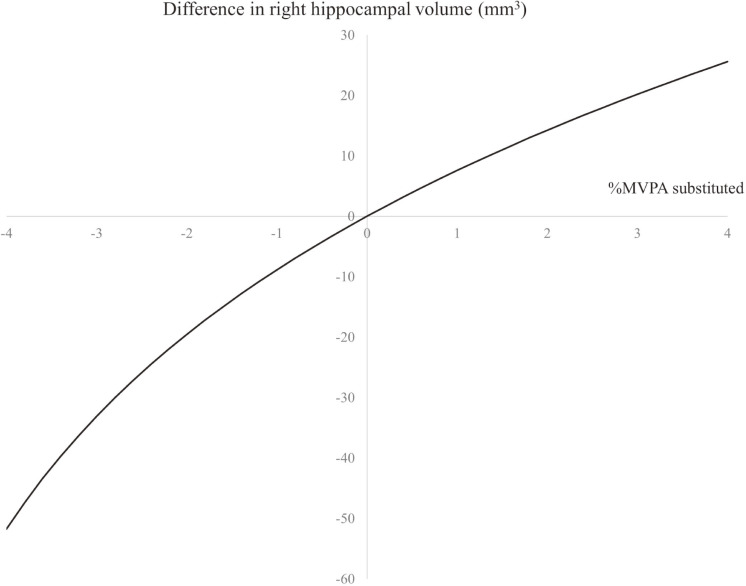
Difference in right hippocampal volume with reallocation of MVPA Analyses were adjusted for sex, age, intracranial volume, education, body mass index, smoking status, alcohol use, and medication for hypertension, dyslipidemia, and diabetes. The reallocation of MVPA expended the remaining activities equally. Mean composition (%/day): sedentary behaviors = 50.5%, light-intensity physical activity = 44.9%, moderate-to-vigorous physical activity = 4.6%. MVPA, moderate-to-vigorous physical activity.

Figure 1 shows the predicted difference in right hippocampal volume with the reallocation of MVPA, after adjustment for potential confounding factors. If a proportion of MVPA decreased by 3% from the mean proportion of MVPA, such as a 4.6% MVPA change to 1.6%, the right hippocampal volume was predicted to be 33.1 mm^3^ smaller (eg, the mean accelerometer wear time in this study was 887.5 min/day; in this case, a 3% decrease translates to a decrease of 26.6 min/day). If a proportion of MVPA increased by 3% from the mean proportion of MVPA, such as a 4.6% MVPA change to 7.6%, the right hippocampal volume was predicted to be 20.2 mm^3^ larger.

## DISCUSSION

In our sample of randomly recruited community-dwelling older adults, the proportion of time spent engaging in MVPA relative to other behaviors (SB and LPA) was favorably associated with right hippocampal volume, even when time spent in other behaviors was taken into account. However, no association was observed between the relative proportion of time spent in MVPA and left hippocampal volume, and neither the relative proportion of time spent in SB nor LPA displayed any association with left or right hippocampal volume. This study has added novel evidence to the emerging field of research regarding the relationship between physical activity and hippocampal volume in community-dwelling older adults.

Hippocampal atrophy has not only been described under pathological conditions, but also in healthy aging.^33^^,^^34^ A meta-analysis estimated the average yearly rate of hippocampal atrophy to be 1.4% in healthy aging.^33^ Further, a recent United Kingdom study of more than 19,700 men and women, aged 45–80 years, reported that age-related hippocampal atrophy accelerates around 65 years of age and that the relationship between hippocampal volume and age had a negative linear relationship among the older.^34^ This study aimed to determine the association between hippocampal volume and physical activity, considering the possibility that physical activity could be a factor inhibiting hippocampal atrophy. Here, the relative proportion of time spent in MVPA, compared to the other two activities, was significantly associated only with right hippocampal volume. Specifically, if a proportion of MVPA decreased by 3% from the mean proportion of MVPA, which is 4.6%, the right hippocampal volume was predicted to be 33.1 mm^3^ smaller. This hippocampal volume loss is at a percentage comparable to approximately 0.9% of the mean right hippocampal volume in this study (3,687.7 mm^3^). A previous longitudinal study used questionnaires to survey older Americans who had no cognitive function impairment and found that time spent walking was positively associated with greater total hippocampal volumes 9 years later.^4^ Although only a few studies have demonstrated a relationship between LPA and hippocampal volume, a cross-sectional study performed with Japanese community-dwelling older adults with MCI revealed that total hippocampal volume was associated only with MVPA and not with LPA.^8^ Additionally, Migueles et al^10^ reported that MVPA was associated with greater right hippocampal volume among children with obesity, although no association was observed between MVPA and left hippocampal volume, and neither SB nor LPA displayed any association with either left or right hippocampal volume. Our results are consistent with those of previous studies and provide further evidence supporting the finding that MVPA is associated with hippocampal volume, whereas LPA is not. Although LPA makes a much larger contribution to energy expenditure than MVPA in the older population,^35^ MVPA may be necessary to maintain hippocampal volume.

A previous meta-analysis revealed that aerobic exercise significantly increases right but not left hippocampal volume.^36^ In the present study, the hippocampal volume was divided into the left and right sides, and the relationship between active behaviors and individual volumes was analyzed separately. Our results indicate that the relative proportion of time spent in MVPA, compared to the other activities, is favorably associated only with right hippocampal volume. There is evidence that hippocampal volume is asymmetrical, with the right hippocampus having a larger volume than the left, although the mechanism for this difference has not been identified.^37^ Further, asymmetry has been observed between the left and right hippocampus of adult mice in terms of the distribution of N-methyl-d-aspartate receptor GluRepsilon2 subunits.^38^ This indicates that the hippocampus is also an asymmetrical brain structure from the perspective of molecular morphology. Additionally, since it has been reported that environmental influences may affect the hippocampus asymmetrically,^39^ MVPA may also affect the volumes of the left and right hippocampus differently.

Several potential mechanisms have been proposed for how MVPA could affect hippocampal volume. Animal studies indicate that aerobic exercise increases levels of brain-derived neurotrophic factor (BDNF) and other growth factors,^40^ as well as cerebral blood volume,^41^ which are known to promote neurogenesis. Among human participants, the levels of BDNF in the blood increase with aerobic exercise, leading to increased hippocampal volume.^42^ MVPA may induce neurogenesis in the right hippocampus through the same mechanism as aerobic exercise.

This study also has several limitations. First, the greatest limitation is that this study is a cross-sectional design, which prevents us from inferring any causal relationship. Second, the response rate of participants was relatively low. Accelerometry responders tended to be healthier than the non-responders, and the possibility of selection bias should, therefore, be taken into account.^43^ Third, few participants spent time in intense physical activity (vigorous physical activity [VPA]), making examination of the relationship between VPA and hippocampal volume impossible.

Despite these limitations, this study improves upon the limitations of previous studies in that they: (1) included only volunteer participants, (2) used questionnaires for measuring physical activity, and did not measure intensity-specific physical activity, and (3) did not take the co-dependence of various intensities of physical activity into account during statistical analyses. To the best of our knowledge, this study is the first to explore associations between activity behaviors and hippocampal volume among randomly recruited community-dwelling older adults, after co-dependence of time spent was explicitly considered. Our results provide further evidence to support the hypothesis that MVPA is favorably associated with right hippocampal volume among community-dwelling older adults.

In conclusion, among community-dwelling older adults, objective measurements of the relative proportion of time spent engaging in MVPA, compared to SB and LPA, was favorably associated with right hippocampal volume. Contrastingly, in models controlling for the proportion of time spent engaging in other behaviors, SB and LPA were not associated with hippocampal volume. Accordingly, spending more time engaging in MVPA than other behaviors may have beneficial effects on right hippocampal volume. The promotion of health through MVPA targeted at community-dwelling older adults may be effective for increasing their right hippocampal volume and therefore preserving their cognitive function.
